# Construction and application of an evidence-based prevention and management protocol for postoperative hypoxemia in elderly patients with hip fractures

**DOI:** 10.3389/fmed.2025.1667172

**Published:** 2026-01-13

**Authors:** Yu Zhang, Xi Chen, Yingqi Zhang, Zhirong Ma, Lili Zhao, Haijiao Zhang

**Affiliations:** 1 Department of Orthopedic Surgery, People’s Hospital of Ningxia Hui Autonomous Region (People’s Hospital of Ningxia Hui Autonomous Region, Ningxia Medical University), Yinchuan, China; 2 Nursing Department, People’s Hospital of Ningxia Hui Autonomous Region (People’s Hospital of Ningxia Hui Autonomous Region, Ningxia Medical University), Yinchuan, China

**Keywords:** hip fracture, hypoxemia, prevention and management, evidence-based practice, protocol development, postoperative complications, elderly patients, oxygen saturation

## Abstract

**Objective:**

To develop an evidence-based practice protocol for the prevention and management of postoperative hypoxemia in elderly patients with hip fracture and to evaluate its clinical effectiveness.

**Methods:**

Utilizing evidence-based nursing methodology, we systematically retrieved, appraised, and synthesized the best available evidence regarding the prevention and management of postoperative hypoxemia in this patient population. Guided by a knowledge translation framework, an evidence-based practice protocol was developed. Elderly hip fracture patients undergoing surgery at a tertiary hospital in Ningxia were enrolled from January-April 2024 (baseline/pre-implementation group, *n* = 52) and June-September 2024 (post-implementation group, *n* = 52). Peripheral oxygen saturation (SpO_2_) was measured at key time points: upon entering the operating room, upon returning to the ward, and on postoperative days (POD) (1) and (2). Comparisons were made between groups regarding SpO_2_ changes, incidence of hypoxemia (SpO_2_ < 90%), and healthcare professionals’ (HCPs) knowledge scores.

**Results:**

Repeated-measures ANOVA revealed significant main effects of time (*F* = 18.177, *p* < 0.001) and group (*F* = 38.818, *p* < 0.001), as well as a significant time*group interaction (*F* = 29.865, *p* < 0.001) on SpO_2_. Multivariate ANOVA showed significantly higher SpO_2_ in the post-implementation group compared to the pre-implementation group on POD1 (*F* = 18.870, *p* < 0.001) and POD2 (*F* = 205.270, *p* < 0.001). The incidence of hypoxemia was significantly reduced in the post-implementation group on POD1 (1.92% vs. 13.46%, χ^2^ = 4.875, *p* = 0.027) and POD2 (0.00% vs. 19.23%, χ^2^ = 11.064, *p* = 0.001). Additionally, HCPs’ knowledge scores significantly increased following protocol implementation (91.54 ± 4.90 vs. 73.08 ± 6.35, *t* = 16.59, *p* < 0.001).

**Conclusion:**

The evidence-based protocol for preventing and managing postoperative hypoxemia effectively enhanced clinical decision-making, improved postoperative SpO_2_ levels, reduced hypoxemia incidence, and enhanced HCPs’ knowledge. These findings support the protocol’s feasibility and effectiveness for clinical implementation in elderly hip fracture patients.

## 1 Introduction

Hypoxemia is a critical postoperative complication that profoundly influences clinical outcomes and impedes the rapid and uneventful recovery of surgical patients (1). Severe hypoxemia can precipitate life-threatening events, including cardiac arrhythmias, hemodynamic instability, delayed wound healing, and extreme cases, cardiac arrest or death (2). Epidemiological studies report that approximately 15.5% of elderly patients with hip fracture experience perioperative hypoxemia (3), a condition closely linked to increased risks of pulmonary complications, multi-organ failure, intensive care unit (ICU) admission, and mortality (4, 5). Given these serious consequences, early detection and proactive management of hypoxemia are paramount in this vulnerable population. However, hypoxemia often remains underrecognized, primarily due to its subtle clinical presentation in mild to moderate cases, which can be easily obscured by common postoperative symptoms such as abdominal distension, pain, chest tightness, and anxiety. Moreover, a subset of patients may not exhibit hallmark signs like chest tightness or cyanosis (6), further complicating timely diagnosis and intervention during the perioperative period in elderly hip fracture patients.

The Expert Consensus on Clinical Practice for Perioperative Nursing of Elderly Hip Fracture Patients (2023 Edition) (7) recommends routine periodic monitoring of peripheral oxygen saturation (SpO_2_) from admission through the entire hospitalization period, with supplemental oxygen therapy and continuous monitoring initiated upon detection of hypoxemia. However, the lack of standardized protocols and limited high-quality evidence have impeded the widespread adoption of routine SpO_2_ monitoring in clinical settings. To address this critical gap, the present study systematically reviewed and appraised existing evidence regarding perioperative hypoxemia prevention strategies in elderly hip fracture patients. Based on this synthesis, we developed an evidence-based intervention protocol and conducted clinical implementation research aimed at effectively preventing postoperative hypoxemia in this high-risk population.

## 2 Materials and methods

### 2.1 Development of the clinical practice protocol

#### 2.1.1 Identification of clinical evidence-based practice questions

The clinical evidence-based practice questions were identified by integrating clinical experience with the PIPOST framework (8), which encompasses the following components: Population (P): Elderly patients with hip fractures; Intervention (I): Hypoxemia prevention and management protocol; Professionals (P): Orthopedic surgeons and nurses; Outcomes (O): Healthcare providers’ knowledge and awareness rates, postoperative SpO_2_ fluctuations, and incidence of hypoxemia; Setting (S): Orthopedic center of a tertiary hospital in Ningxia; Type of Evidence (T): Clinical decision-making, guidelines, expert consensus, evidence summaries, and systematic reviews.

#### 2.1.2 Establishment of a multidisciplinary evidence-based practice team

The evidence-based practice team comprised members with systematic training in evidence-based methodology, including: Three nursing researchers responsible for evidence translation, protocol development, data collection and analysis; One chief of trauma orthopedics and one resident physician who provided clinical guidance for evidence implementation and operational supervision; One nurse manager and two clinical nurse specialists from the trauma orthopedics ward overseeing protocol execution and quality control.

#### 2.1.3 Evidence acquisition

Guided by the 6S evidence pyramid model, we systematically searched for evidence on hypoxemia prevention and management in hip fracture patients across multiple databases, including The Cochrane Library, the National Institute for Health and Care Excellence (NICE) website, China National Knowledge Infrastructure (CNKI), Wanfang Database, and VIP Database. The search period extended from the inception of each database to March 2024. A total of seven publications were included, comprising four expert consensus documents and three clinical guidelines.

#### 2.1.4 Evidence-Based protocol formulation

Three independent evaluators appraised the included expert consensus documents and clinical guidelines using the Professional Consensus Quality Assessment Tool and AGREE II instrument, respectively. Through this rigorous evaluation, six best-practice recommendations were synthesized.

Subsequently, a multidisciplinary panel meeting was convened, consisting of two trauma orthopedic surgeons, five nursing specialists, one anesthesiologist, and one pulmonologist. All panel members held associate senior titles or higher and possessed over 8 years of clinical experience. The panel critically evaluated the evidence across four key domains: feasibility, appropriateness, clinical significance, and effectiveness. During the discussion, specific operational modifications were proposed and deliberated for Recommendations 3–5, including: Intervention stratification: Development of tiered management protocols based on SpO_2_ monitoring thresholds. Respiratory training optimization: Refinement of pulmonary rehabilitation exercises, specifying modalities, frequency (three times daily), and intensity (progressive increase to 30% of maximal inspiratory pressure) in accordance with Recommendation 5. The final six validated indicators were implemented into the clinical practice protocol (see Table 1).

**TABLE 1:** Evidence synthesis and appraisal methodology for hypoxemia prevention and management in elderly hip fracture patients.

Original evidence description	Audit indicator	Auditor	Method
1. Routine use of pulse oximetry reduces hypoxemia incidence.	1. Pulse oximetry is employed for continuous monitoring of SpO_2_.	Nurse	Observation
2. Routine SpO_2_ check on admission.	2. SpO_2_ measurement should be performed on the day of patient admission.	Nurse	Observation
3. Periodic monitoring during hospitalization.	3. Monitoring frequency is stratified based on admission SpO_2_ levels: • SpO_2_ > 95%: every 8 h (Q8h) • 90% ≤ SpO_2_ ≤ 95%: every 6 h (Q6h) • 86% < SpO_2_ < 90%: every 1 h (Q1h)	Nurse	Record review
4. Continuous monitoring if hypoxemia risk; oxygen therapy for hypoxemia.	4. Oxygen therapy is administered according to SpO_2_ values: • For 90% ≤ SpO_2_ ≤ 95%: nasal cannula oxygen for 2 hours post-meals and continuous overnight oxygen supplementation after 22:00. • For 86% < SpO_2_ < 90%: pulmonology consultation is recommended; continuous nasal oxygen is provided, with consideration of mask oxygen or high-flow oxygen therapy as clinically indicated.	Nurse	Record review
5. Respiratory training combined with O_2_ therapy effectively reduces hypoxemia.	5. Respiratory training protocol: • Balloon Blowing Exercise: Inflate 2–3 balloons, three times daily. The patient inhales deeply over 1–2 s, holds breath for 2–3 s, then exhales slowly and steadily into balloon over 3–5 s until the balloon diameter reaches 5–20 cm. • *Incentive Spirometer (800–1,000 mL capacity):* Perform 2–3 sets, three times daily, in semi-Fowler’s or sitting position. Each set consists of three deep breaths. The patient seals lips around the mouthpiece, inhales slowly to elevate the ball or float to the target volume without exceeding the flow limit, holds breath for 2–3 s, then exhales slowly. Rest intervals are allowed between sets.	Nurse	Interview
6. Intensify SpO_2_/blood gas monitoring and respiratory training within 3 days post-op.	6. A designated nurse is responsible for daily SpO_2_ monitoring and supervises of respiratory training adherence.	Nurse	Record review

### 2.2 Application and effect evaluation of the evidence-based practice program

#### 2.2.1 Data and methods

This study used patients’ initial oxygen saturation measurement as the primary outcome indicator. The sample size was calculated based on the formula for comparing the means of two independent samples: *n* = (μ_α_ + μ_β_)^2^ × 2σ^2^/δ^2^, set the test level of α = 0.05, β = 0.1. According to the standard normal distribution table, the corresponding values are μ_α_ = 1.96, and μ_β_ = 1.282. Taking the patients’ first oxygen saturation monitoring value as the main index, the investigated σ = 5.99, the proposed post-intervention increase in oxygen saturation values was 4. Substituting these values yielded a required sample size of *n* = 45 per group. Considering a 15% margin for potential attrition or calculation error, the final sample size was adjusted to 52 participants per group, resulting in a total sample size of 104.

#### 2.2.2 Study subjects

A quasi-experimental, pre-post study design was utilized. Participants were assigned to groups based on the timing of their surgery: The pre-implementation (control) group consisted of consecutive eligible patients admitted between January and April 2024, who received routine standard care. The post-implementation (intervention) group consisted of consecutive eligible patients admitted between June and September 2024, who were managed according to the new evidence-based protocol. Inclusion criteria: ➀ age ≥ 65 years; ➁ Confirmed diagnosis of hip fracture; ➂ surgical intervention performed within 24 h of admission; ➃ patients who voluntarily agreed to participate in this study. Exclusion criteria: ➀ fractures caused by metastatic tumor; ➁ presence of rib fracture; ➂ intractable hypoxemia; ➃ severe cardiopulmonary diseases or cerebral hemorrhage; ➄ time of injury ≥ 2 weeks; ➅ operation duration ≥ 4 h; ➆ postoperative transfer to intensive care unit (ICU); ➇ American Society of Anesthesiologists (ASA) physical status classification grade IV or higher. This study was reviewed and approved by the Ethics Committee of Ningxia Hui Autonomous Region People’s Hospital (approval number: 2023-NZR-105). Informed consent was obtained from all participants prior to enrollment.

#### 2.2.3 Standardization of preoperative care

To control for potential confounding from variations in preoperative management, all patients in both the pre- and post-implementation groups were treated in accordance with the institution’s standardized clinical pathway for enhanced recovery after surgery (ERAS) in hip fracture patients.

### 2.3 Clinical introduction of evidence-based practice programs

#### 2.3.1 The team adopted a brainstorming method to analyze potential facilitating factors and obstacles to implementation.

##### 2.3.1.1 Facilitating factors

➀ Since 2018, the Department of Traumatology and Orthopaedics has been actively promoting the concept of accelerated rehabilitation surgery, and healthcare personnel demonstrate a high level of receptiveness to new knowledge and practices; ➁ The Department of Traumatology and Orthopaedics serves as a provincial quality control center, providing a strong institutional foundation for evidence-based initiatives; ➂ The department director is highly supportive of the program, fostering effective collaboration and tacit cooperation among healthcare staff; ➃ The nursing team includes two postgraduate students with robust scientific research capabilities, enhancing the team’s capacity for evidence-based practice and quality improvement.

##### 2.3.1.2 Barrier factors

➀ Health care personnel exhibit insufficient attention to hypoxemia and possess limited knowledge regarding its prevention and management; ➁ There is a lack of standardization protocols and processes for hypoxemia prevention and management; ➂ Insufficient availability of equipment for continuous oxygen saturation monitoring; ➃ The patients population primarily consists of elderly individuals with poor compliance and limited acceptance of new knowledge and interventions.

##### 2.3.1.3 Establishment of a research team

A dedicated hypoxemia prevention and management team for elderly hip fracture patients was established, comprising eight members with clearly defined roles: Two orthopedic surgeons, one respiratory surgeon, and one anesthesiologist, primarily responsible for the development, implementation, and ongoing adjustment of the evidence-based practice program. Two ward nurses tasked with program execution, real-time feedback collection, and outcome evaluation. One nurse assigned to data collection and organization. The head nurse overseeing overall supervision, quality control, and feedback management to ensure adherence to protocols and continuous improvement.

#### 2.3.2 Practice change

##### 2.3.2.1 Establishment of hypoxemia prevention and management processes and quality control methods in the ward, and preparation of nursing implementation checklists

Based on the evidence-based practice program, a standardized management protocol for perioperative hypoxemia prevention in elderly hip fracture patients was developed, accompanied by a detailed clinical pathway. Correspondingly, a nursing measures implementation was prepared to guide daily care activities. Each day, the designated nurse monitors patients’ oxygen saturation and respiratory function training according to the clinical pathway, documents the implementation status, records relevant clinical values, and marks the corresponding nursing interventions on the checklist. Two quality control specialists are assigned to conduct daily audits of the implementation and documentation, providing timely feedback and recommending corrective actions upon identification of any issues. Additionally, the nurse manager performs random inspections twice weekly to conduct summary analysis and drive continuous quality improvement.

##### 2.3.2.2 Strengthening systematic training and assessment

➀ Business learning: The project leader introduces the origin, evidence basis, detailed implementation plan, and management methods of the hypoxemia prevention program, organizing structured learning sessions for nurses to ensure comprehensive understanding. ➁ Visual aids and learning materials: The hypoxemia prevention and management protocol is printed on cards and displayed at each patient’s bedsite for easy reference. Additionally, relevant knowledge manuals are produced and made available to nurses for timely consultation. Training videos and question banks related to the program are developed and distributed to workgroups to facilitate continuous learning. ➁ Scenario simulation: Monthly scenario-based exercises on hypoxemia prevention and management are conducted to identify potential deficiencies or loopholes in the management process, enabling iterative improvements. ➂ Regular assessment: Knowledge retention and program adherence are reinforced through daily morning meeting quizzes and weekly evaluations focusing on key components and specific content of the program.

##### 2.3.2.3 Implementation of diversified health education

➀ Teach-back method: Utilize the Teach-Back approach to provide practical guidance to patients and their caregivers. After instruction, patients and caregivers are asked to demonstrate their understanding and mastery of the content. Nurses then provide correction and supervision as needed. Additionally, multidisciplinary education involving doctors, nurses, and rehabilitation therapists is conducted to promote early mobilization and activity from multiple perspectives. ➁ Educational videos: Develop and produce popular science videos covering respiratory exercise, oxygen therapy, and hypoxemia prevention. These videos are regularly played using mobile health education units and ward televisions to enhance patient and caregiver understanding.

### 2.4 Evaluation methods

#### 2.4.1 Oxygen saturation measurement and quality control

Peripheral oxygen saturation (SpO_2_) was measured using a Yuwell YX306 finger-clip pulse oximeter (Medical Device Registration Certificate No.: Su Zhun Zhun 20172071070). To minimize the impact of common interfering factors and ensure the acquisition of reliable data, a strict standardized protocol was implemented as follows: (1) Patient Preparation and State: Measurements were taken after the patient had been resting quietly in a supine or semi-Fowler’s position for at least 10 min in a non-oxygenated state. (2) Sensor Placement and Site Selection: The sensor was placed on the clean, warm, and unscarred distal phalanx of the index finger. Fingers with nail polish, artificial nails, or obvious deformities were avoided. The chosen site was checked for adequate local perfusion. This site was selected due to its widespread clinical use and practicality for repeated measurements. (3) Stabilization and Recording: The displayed SpO_2_ value was recorded only after it had stabilized along with the waveform for a continuous period of 15–20 s. Transient fluctuations were ignored. (4) Environmental Controls: Strong ambient light sources (e.g., direct sunlight) were shielded from the sensor to prevent optical interference. This comprehensive quality control protocol was consistently applied by trained nursing staff to all measurements across both study groups, ensuring the comparability and reliability of the SpO_2_ data.

#### 2.4.2 Incidence of hypoxemia

Hypoxemia was defined according to the standard criteria (9): under room air conditions, SpO_2_ < 90% was considered indicative of hypoxemia.

#### 2.4.3 Knowledge of assessment of medical and nursing staff

A test was developed to evaluate the knowledge of medical and nursing staff regarding the hypoxemia prevention and management program. To ensure its content validity and clinical relevance, the initial draft was reviewed by a multidisciplinary expert panel comprising two senior orthopedic surgeons, one pulmonologist, and three nursing experts. The panel evaluated the questions for clarity, appropriateness, and comprehensiveness in covering the key components of the protocol. Their feedback was used to refine the wording and content of the questionnaire. The test covers four key areas: concepts, classification, preventive measures, and emergency treatment. It consists of 20 questions, each worth 5 points, with a total score of 100 points. A higher score indicates a better mastery of the relevant knowledge. The questionnaire was pilot-tested with a group of 10 nurses not involved in the main study to identify any ambiguities in the questions or options, and minor revisions were made accordingly.

### 2.5 Statistical methods

Statistical analysis was performed using SPSS 23.0 software. Continuous variables conforming to a normal distribution were expressed as mean ± standard deviation (SD), and comparisons between two groups were conducted using the independent samples *t*-test. Continuous variables not conforming to a normal distribution were expressed as median and interquartile range (IQR), with group comparisons performed using the Mann-Whitney U-test. Categorical variables were expressed as frequency and percentage, and differences between groups were analyzed using the chi-square test. For repeated-measures data, two-way ANOVA was applied if Mauchly’s test of sphericity was satisfied; otherwise, the Greenhouse-Geisser correction was used. In the repeated-measures ANOVA results, if no interaction effect between time and treatment factors was observed, the main effects test was used to evaluate the effect of treatment. If an interaction effect existed, simple effects analysis were conducted to explore the differences.

## 3 Results

### 3.1 Baseline data of patients

A total of 104 elderly patients with hip fracture were included in the study, with 52 patients assigned to each group. Comparison of baseline characteristics between the two groups showed no statistically significant differences (*p* > 0.05), as detailed in Table 2.

**TABLE 2:** Comparison of baseline characteristics between groups.

Variable	Pre-implementation group (*n* = 52)	Post-implementation group (*n* = 52)	Statistic (*t/*χ^2^)	*p*
Gender, n (Male/Female)	17/35	21/31	0.663	0.415
ASA grade, n (P2/P3)	7/45	10/42	−0.790	0.430
Age, years (Mean ± SD)	77.19 ± 8.39	79.13 ± 7.71	1.229	0.222
Surgery duration, hours (mean ± SD)	3.01 ± 0.55	2.87 ± 0.44	−1.386	0.169
Admission NRS (Mean ± SD)	3.90 ± 1.67	3.77 ± 1.44	−0.440	0.660
Admission ADL (Mean ± SD)	40.09 ± 8.43	43.13 ± 10.96	1.580	0.120
Admission SpO_2_ (%, Mean ± SD)	93.31 ± 3.84	94.04 ± 3.48	1.017	0.251
Admission Hypoxemia, n (%)	7 (13.46%)	6 (11.53%)	0.088	0.767
Concomitant diseases—respiratory diseases, n (%)	10 (19.20%)	13 (25.00%)	0.502	0.478
Concomitant diseases—cardiovascular disease, n (%)	26 (50.00%)	33 (63.50%)	1.919	0.166
Concomitant diseases—metabolic and endocrine diseases, n (%)	11 (21.20%)	10 (19.20%)	0.060	0.807
Concomitant diseases—neurological diseases, n (%)	6 (11.50%)	10 (19.20%)	1.182	0.277
Concomitant diseases—malnutrition, n (%)	15 (28.80%)	22 (42.30%)	2.056	0.152

ADL, Activities of Daily Living; NRS, Numeric Rating Scale (Pain); ASA, American Society of Anesthesiologists Physical Status Classification.

### 3.2 Main effect test of oxygen saturation levels

An analysis was conducted on the oxygen saturation levels of patients in the two groups at different time points before and after the implementation of evidence-based practice. Mauchly’s test of sphericity indicated that the variance-covariance matrix of oxygen saturation measurements across time points did not meet the sphericity assumption (χ^2^ = 56.674, *p* < 0.001), necessitating correction of the test statistics using the Greenhouse-Geisser method.

**FIGURE 1:**
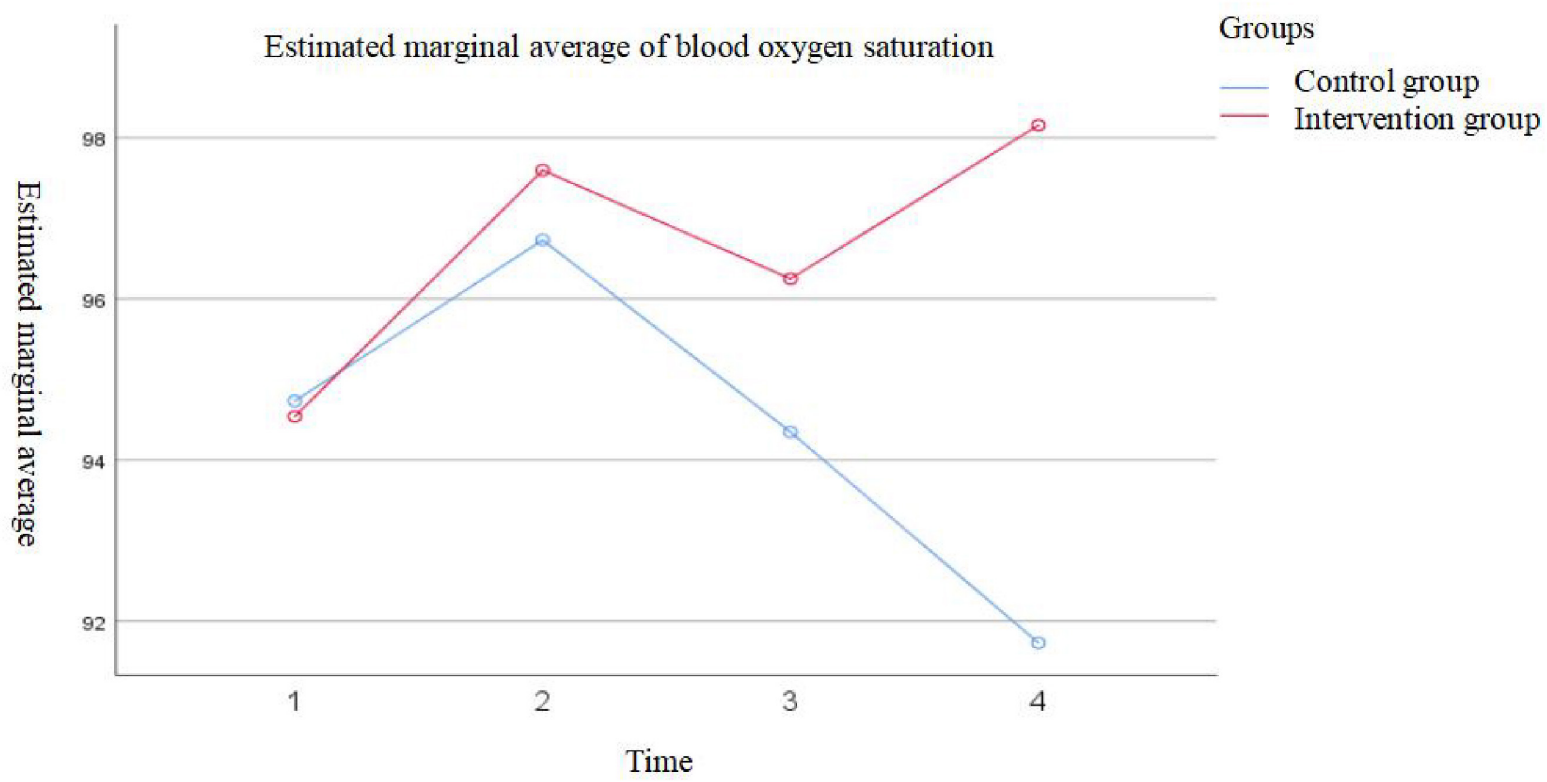
Group-based effect analysis.

**FIGURE 2:**
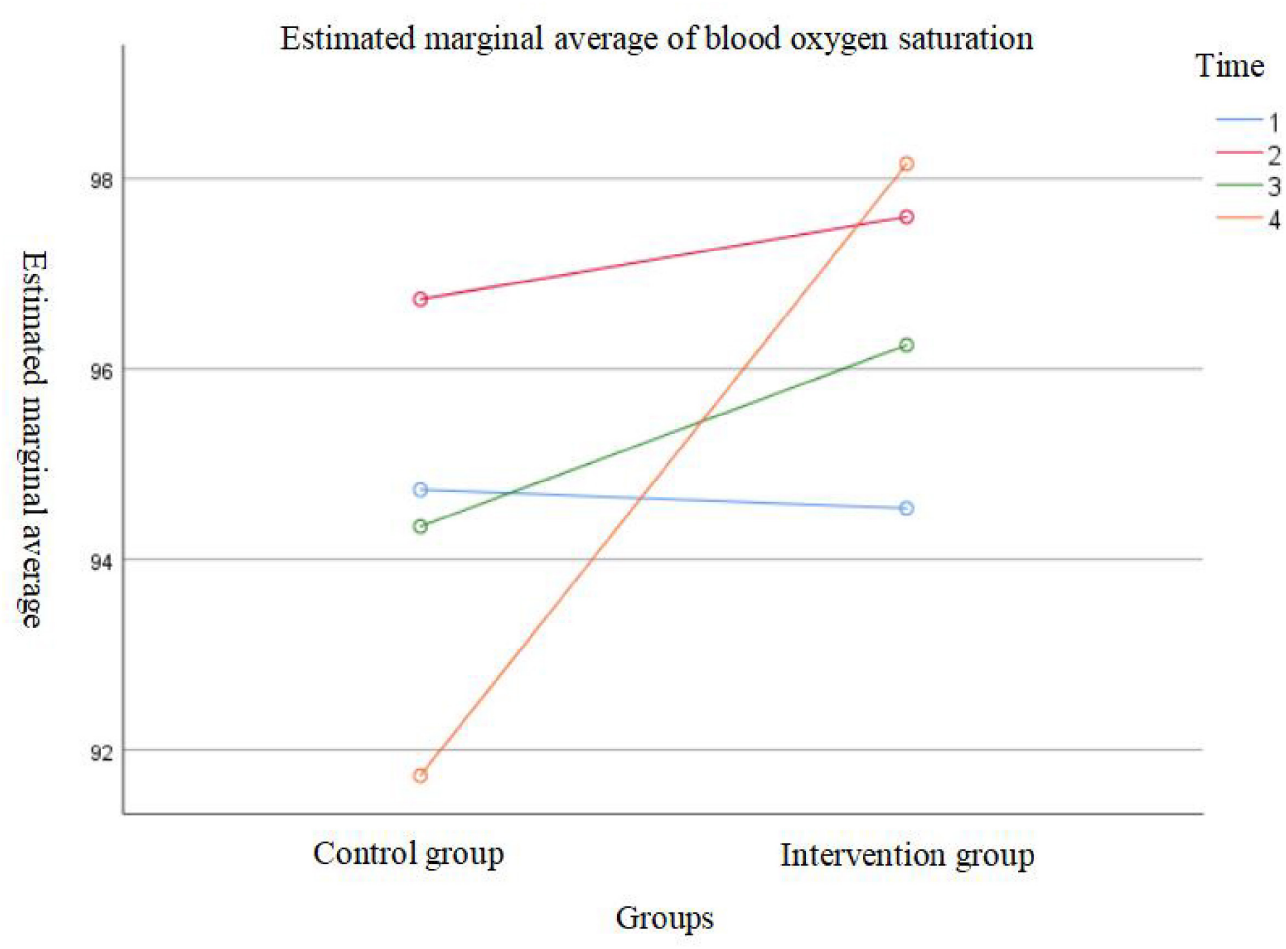
Effect analysis based on time points.

The results showed a significant main effect of time on oxygen saturation levels (*F* = 18.177, *p* < 0.001), indicating that oxygen saturation changed significantly over time in both groups. The between-group effect was also statistically significant (*F* = 38.818, *p* < 0.001), suggesting a significant difference in oxygen saturation levels between the groups before and after evidence-based practice implementation. Additionally, the interaction effect between time and group was significant (*F* = 29.865, *p* < 0.001), indicating that the pattern of oxygen saturation changes over time differed between the two intervention protocols. Therefore, further simple effect analysis were performed separately for each group. Detailed data are presented in Table 3 and Figures 1, 2.

**TABLE 3:** Repeated measures analysis of variance on oxygen saturation levels at different time points in two patient groups.

Pre- and post-intervention implementation (*n* = 104)						
Indicator	Time effect		Between—group effect		Interaction effect	
	F	p	F	p	F	p
SpO_2_	18.177	< 0.001	38.818	< 0.001	29.865	< 0.001

### 3.3 Simple effect test of oxygen saturation levels

After adjusting the significance level using the Bonferroni correction (α’ = 0.008), the comparison of SpO_2_ levels at different time points in the post-evidence-based practice group showed that: SpO_2_ levels before admission were significantly lower than those at the first measurement after surgery and on the second day after surgery; SpO_2_ levels on the first day after surgery were lower than those on the second day after surgery. However, no statistically significant differences were found between SpO_2_ levels before admission and on the first day after surgery, between the first measurement after surgery and the first day after surgery, or between the first measurement after surgery and the second day after surgery (all *p* > 0.05).

In the pre-evidence-based practice group, the comparison showed that: SpO_2_ levels before admission were lower than those at the first measurement after surgery; SpO_2_ levels at the first measurement after surgery were higher than those on the first day after surgery, which were in turn higher than those on the second day after surgery. Nonetheless, no statistically significant differences were observed between SpO_2_ levels before admission and the first measurement after surgery, or between before admission and the first day after surgery (both *p*> 0.05) (see Table 4 for details).

**TABLE 4:** Comparison of perioperative SpO_2_ levels (mean ± SD, %) between groups.

Group	n	Pre-Op	Post-Op return	POD1	POD2	*F* (Time)	*p* (Time)
Post-implementation	52	94.54 ± 4.86	97.60 ± 2.12	96.25 ± 2.12	98.15 ± 1.72	20.97	<0.001
Pre-implementation	52	94.73 ± 3.64	96.73 ± 3.11	94.35 ± 2.34	91.73 ± 2.74	49.92	<0.001
*F* (Group)		0.05	2.76	18.87	205.27		
*p* (Group)		0.82	0.10	<0.001	< 0.001		

*F-* and *p*-value for time effect within group (One-way RM-ANOVA). *F* and *p*-value for group effect at each time point (Between-subjects effect from RM-ANOVA simple effects). POD, postoperative day.

Multivariate analysis of variance (MANOVA) results indicated that SpO_2_ levels on the first day and second day after surgery in the post-evidence-based practice group were significantly higher than those in the pre-evidence-based practice group (*F* = 18.87, *p*< 0.001; *F* = 205.27, *p*< 0.001). However, no significant differences were found between the two groups at the time points before admission and at the first measurement after surgery (*F* = 0.05, *p* = 0.82; *F* = 2.76, *p* = 0.10) (see Table 4 for details).

### 3.4 Comparison of postoperative hypoxemia incidence between post-evidence-based practice and pre-evidence-based practice

On the first postoperative day, the incidence of hypoxemia in the post-evidence-based practice group (1.92%) was significantly lower than that in the pre-evidence-based practice group (9.61%). While the pre-evidence-based practice group exhibited a significant upward trend in hypoxemia incidence, the post-evidence-based practice group showed a significant decrease. These differences were statistically significant (*p* < 0.05) (see Table 5 for details).

**TABLE 5:** Comparison of postoperative hypoxemia incidence between groups (*n* = 104).

Time point	Group	Hypoxemia Cases (n, %)	Non-Hypoxemia cases (n, %)	χ^2^	*p*
Postoperative day 1	Post-implementation (*n* = 52)	1 (1.92%)	51 (98.08%)	4.875	0.027
	Pre-implementation (*n* = 52)	7 (13.46%)	45 (86.54%)		
Postoperative day 2	Post-implementation (*n* = 52)	0 (0.00%)	52 (100.00%)	11.064	0.001
	Pre-implementation (*n* = 52)	10 (19.23%)	42 (80.77%)		

### 3.5 Comparison of medical staff’s knowledge awareness scores on hypoxemia management before and after evidence-based practice

The knowledge awareness score of medical staff regarding hypoxemia management after the implementation of evidence-based practice (91.54 ± 4.90) was significantly higher than that before implementation (73.08 ± 6.35), with the difference being statistically significant (*t* = 16.59, *p* < 0.05).

## 4 Discussion

This study successfully developed and implemented a structured, evidence-based protocol for the prevention and management of postoperative hypoxemia in elderly hip fracture patients. The primary objective was to evaluate its clinical effectiveness, and our findings robustly demonstrate that the protocol was not only feasible but also profoundly effective. The most significant outcomes—the dramatic reduction in hypoxemia incidence and the sustained improvement in postoperative oxygen saturation—underscore the value of bridging evidence into practice.

### 4.1 Feasibility and safety

The protocol is grounded in evidence-based SpO_2_ monitoring using pulse oximetry, which exploits the distinct light absorption characteristics of oxygenated and deoxygenated hemoglobin (10). Modern pulse oximeters provide SpO_2_ measurements that closely correlate with arterial oxygen saturation (SaO_2_), with an error margin of less than 2% (11), making them reliable, non-invasive, and practical tools for continuous monitoring and timely clinical intervention (12, 13). A key strength of this protocol lies in its stratified approach, whereby monitoring frequency and intervention intensity, such as oxygen therapy and respiratory training, are dynamically tailored according to patients’ SpO_2_ levels. This individualized strategy effectively maintained SpO_2_ within safer ranges and significantly reduced the incidence of hypoxemia, thereby demonstrating the protocol’s feasibility and safety for clinical application.

### 4.2 Effectiveness in improving SpO_2_ and reducing hypoxemia

#### 4.2.1 Superiority of the stratified protocol over conventional care

The most critical finding of our study is the dramatic reduction in hypoxemia incidence, from 13.46% (POD1) and 19.23% (POD2) in the control group to 1.92 and 0.00%, respectively, in the intervention group. This success can be attributed to the protocol’s proactive and dynamic design, which moves beyond conventional care that often discontinues monitoring after POD1 if SpO_2_ > 90% (7). Our approach is consistent with recent international literature; for instance, a PRIME-AIR study protocol by Fernandez-Bustamante et al. concluded that bundled interventions, which combine monitoring, oxygen therapy, and respiratory training, are most effective at preventing postoperative pulmonary complications (14). Our protocol operationalizes this “bundle” concept into a precise, actionable clinical pathway. The significant “Group” effect and “Time × Group” interaction in our ANOVA further confirm that the intervention itself exerted a unique and powerful effect on stabilizing oxygenation over time. Although our study was not specifically designed for formal subgroup analyses by variables such as gender and age, several aspects of our data and analysis address this concern. First, as shown in Table 2, these key baseline characteristics were balanced between the pre-intervention and post-intervention groups. This balance reduces the likelihood that the observed significant differences in hypoxemia incidence and SpO_2_ levels were influenced by these variables. Furthermore, repeated-measures ANOVA revealed a highly significant main effect of “Group” and a significant interaction between “Time × Group.” This indicates that the intervention itself exerts a robust and unique effect on the trajectory of oxygenation over time, an effect that is consistent across the study population. The primary objective of this study was to evaluate the overall efficacy of the bundled protocol; future research with larger sample sizes could help explore the moderating effects of specific patient or surgical factors on treatment outcomes.

#### 4.2.2 Addressing the persistent nature of postoperative hypoxemia

Our data revealed that the highest incidence of hypoxemia in the control group occurred on POD2 (19.23%), a finding that underscores a commonly overlooked clinical window. This pattern aligns with the prospective study by Sun et al., which demonstrated that postoperative hypoxemia is not only common but also persistent, often extending beyond the immediate recovery period (9). Our protocol, by mandating a minimum of three days of dynamic monitoring, directly addresses this persistence and effectively prevents its occurrence. Regarding adverse events attributable to hypoxia in the pre-implementation group, no catastrophic events such as cardiac arrest or death were directly documented during the study period in either group. However, the significantly higher incidence of hypoxemia (SpO_2_ < 90%) observed in the pre-implementation group (13.46% on POD1 and 19.23% on POD2) is itself a cause for grave concern. Extensive literature unequivocally links even transient postoperative hypoxemia to an increased risk of secondary complications, including myocardial injury, delirium, impaired wound healing, and prolonged hospitalization (1, 2, 4). The absence of immediate, life-threatening events in our cohort should not diminish the clinical significance of these findings; rather, it highlights that hypoxemia often operates as a ‘silent’ precursor to more serious morbidity. Therefore, the primary value of our evidence-based protocol lies in its proactive prevention of hypoxemia, thereby mitigating the risk cascade associated with it, rather than merely reacting to its most extreme consequences.

#### 4.2.3 Synergistic effect of combined interventions

The combined use of oxygen therapy and structured respiratory training [e.g., incentive spirometry (15)] was a cornerstone of our protocol. This combination optimally engages respiratory muscles and helps prevent atelectasis. Our results strengthen the existing evidence (16) by demonstrating its efficacy within a comprehensive management strategy for a high-risk population. When compared to national studies, our pre-implementation hypoxemia rate was similar to the 15.5% reported by Liu et al. (3). However, our post-implementation results suggest that the comprehensive implementation strategy—including staff education, visual aids, and quality control—was crucial for achieving outcomes that surpass those of simpler interventions.

### 4.3 Enhancing healthcare professionals’ knowledge

The successful implementation and sustainability of evidence-based interventions depend largely on their feasibility, appropriateness, and perceived value, as well as on effective interdisciplinary collaboration. Our systematic approach – engaging healthcare professionals in the development of the protocol, providing clear clinical pathways and checklists, leveraging technology for training and education, and establishing rigorous quality control mechanisms such as daily monitoring and audits—fostered strong engagement and adherence. This structured, evidence-based quality improvement process significantly enhanced healthcare professionals’ knowledge and understanding of hypoxemia management in this vulnerable patient population, thereby contributing to improved clinical outcomes and sustained practice change.

### 4.4 Limitations and future perspectives

This study has several limitations. First, factors such as the type of anesthesia type (general versus regional), opioid administration, and sedation levels can substantially influence oxygen saturation. However, since all participants in this study received regional anesthesia, the generalizability of the findings may be limited. Second, although we recognized that the type of hip fracture surgery (e.g., arthroplasty vs. internal fixation) might influence the risk of perioperative hypoxemia, this variable was not systematically controlled for in our analysis. However, all patients were managed within the same orthopedic center under standardized anesthesia protocols (regional anesthesia), which may have mitigated some of the variations. Third, SpO_2_ monitoring was limited to the first two postoperative days. Future research should explore the effectiveness of this protocol across different anesthesia types, opioid regimens, and sedation levels, and consider extending SpO_2_ monitoring until patient discharge to further optimize and refine the protocol.

## 5 Conclusion

The evidence-based protocol developed for the prevention and management of postoperative hypoxemia in elderly patients with hip fracture has been proven to be practical, clinically applicable, and effective. It significantly improved postoperative SpO_2_ levels, reduced the incidence of hypoxemia, and enhanced healthcare professionals’ knowledge.

## Data Availability

The raw data supporting the conclusions of this article will be made available by the authors, without undue reservation.
